# Liquefaction of Biomass and Upgrading of Bio-Oil: A Review

**DOI:** 10.3390/molecules24122250

**Published:** 2019-06-17

**Authors:** Shiqiu Zhang, Xue Yang, Haiqing Zhang, Chunli Chu, Kui Zheng, Meiting Ju, Le Liu

**Affiliations:** 1 College of Environmental Science and Engineering, Nankai University, Jinnan District, Tianjin 300350, China; 1120170189@mail.nankai.edu.cn (S.Z.); 2120170638@mail.nankai.edu.cn (X.Y.); 1710731@mail.nankai.edu.cn (H.Z.); chucl@nankai.edu.cn (C.C.); 2 Tianjin Engineering Research Center of Biomass Solid Waste Resources Technology, Nankai University, Jinnan District, Tianjin 300350, China; 3 Analytical and Testing Center, Southwest University of Science and Technology, Mianyang 621010, China; zhengkui@swust.edu.cn

**Keywords:** review, biomass, liquefaction, bio-oil, upgrading

## Abstract

The liquefaction of biomass is an important technology to converse the biomass into valuable biofuel. The common technologies for liquefaction of biomass are indirect liquefaction and direct liquefaction. The indirect liquefaction refers to the Fischer–Tropsch (F–T) process using the syngas of biomass as the raw material to produce the liquid fuel, including methyl alcohol, ethyl alcohol, and dimethyl ether. The direct liquefaction of biomass refers to the conversion biomass into bio-oil, and the main technologies are hydrolysis fermentation and thermodynamic liquefaction. For thermodynamic liquefaction, it could be divided into fast pyrolysis and hydrothermal liquefaction. In addition, this review provides an overview of the physicochemical properties and common upgrading methods of bio-oil.

## 1. Introduction

Fossil fuels, such as coal, oil, and natural gas, are non-renewable resources. They play a vital role in human life and social progress. Although, the total amount of fossil fuels on the earth would meet the needs of human beings for several decades, these non-renewable fossil fuels would eventually run out [1]. In addition, the excessive emission of greenhouse gases (such as CO, CO_2_, NOx, SOx, and CH_4_) into the atmosphere makes the global climate uncontrollable with the consumption of fossil fuels [2]. Hence, the renewable fuels (nuclear energy, solar energy, wind energy, and biomass energy) should be further developed [3,4]. Biomass has a high utilization potential and is one of the most important energy sources of the future.

**Figure 1:**
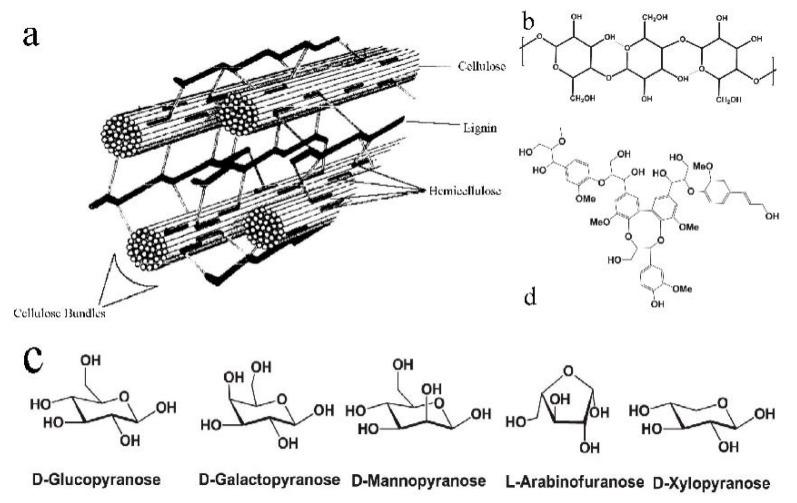
(**a**) Structure of plant cell walls [8]; (**b**) structure of cellulose [9]; (**c**) basic units of hemicellulose [9]; and (**d**) simple structure of lignin [9].

**Figure 2:**
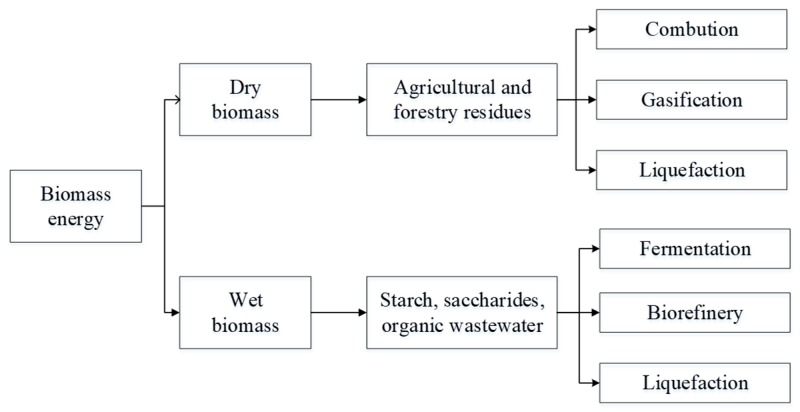
Current conversion technologies of biomass.

Generally, biomass is usually grouped as follows [5,6,7]: (1) Agricultural and forestry residues, (2) herbaceous crops, (3) aquatic and marine biomass, and (4) wastes. Biomass waste means the materials generated in the process of production or consumption of biomass, including wood, straw, animal dungs, and household garbage. Lignocellulose is a complex structure (Figure 1a), which is composed of a mixture of cellulose (Figure 1b), hemicellulose (Figure 1c), lignin (Figure 1d), and inorganic components. Cellulose and hemicellulose are tightly bound to lignin mainly by hydrogen and covalent bonds. Cellulose is usually represented by (C_6_H_10_O_5_)n, and the polymerization degree of the long polysaccharide chain is approximately 10,000, resulting in a high molecular weight (>500,000). Cellulose is formed by the β-1,4 glycosidic linkage of *D*-glucopyranose units. For hemicellulose, the content in dry biomass is usually about 25%, and it is a low degree of amorphous heteropolysaccharide with high degree branching of a straight-chain skeleton. The basic units in hemicellulose are xylan and gulucomannan. Lignin is an amorphous aromatic natural polymer, which is linked primarily via ether bonds with hydroxyl and methoxy groups. The solubility of lignin in water is very low. The main roles of lignin in a plant are as follows: Strengthen their structure, regulate the flow of fluids, protect against microorganisms, and store energy. As we know, biomass energy is the exclusive renewable organic carbon resource to produce liquid fuels. Biomass is the renewable organic materials. While the biomass waste still belongs to the macroscopic category of biomass. According to the statistics, the total amount of biomass waste could reach 2 × 109 t/Y. Nevertheless, only about 40% of biomass waste is used for fuel, building materials, feed, and power generation, and the residual parts are treated in an extensive mode, for instance incineration, landfill, and centralized stacking, resulting in a waste of resources and serious pollution of the environment. Therefore, it is the key component of sustainable development for biomass waste resources. Recently, the mainly technologies of the utilization of biomass resource are physical, thermochemical, and biological. Considering the type of products, it could be divided into gasification, liquefaction, biorefinery, and combustion. Figure 2 shows the current conversion technologies of biomass. For the dry biomass, the primary pathways are combustion, gasification, and liquefaction. Moreover, the products of gasification of biomass could also be conversed to liquid products. For the wet biomass, the primary pathways are hydrolysis fermentation, biorefinery, and liquefaction. The products of biomass conversion are abundant, including syngas, bio-oil, biodiesel, methyl alcohol, ethyl alcohol, etc.

This review paper aims to provide a solution of the current liquefaction technologies of biomass, including indirect liquefaction and direct liquefaction. In indirect liquefaction, we mainly discuss the synthesis pathway of ethyl alcohol and summarize the common catalysts. In direct liquefaction, we discuss the hydrolysis fermentation and thermodynamic liquefaction. In the end, the upgrading technologies of bio-oil have been provided.

## 2. Indirect Liquefaction

### 2.1. Reaction Process

Indirect liquefaction is a promising technology, which is divided into two stages. The first stage is a thermochemical gasification process [10,11]. In this process, the syngas is produced after the raw material reacts with air or steam. In the syngas, the primary substances are CO, CO_2_, H_2_, and H_2_O. The second stage is the well-established Fischer–Tropsch (F–T) process [12]. During the F–T process, the mixture would be used to produce a range of chemicals, including methyl alcohol, dimethyl ether, and ethyl alcohol, while there is little research on the higher alcohols derived from the biomass syngas. The biggest challenges are the design of the novel catalytic reactor for the typically smaller scale of biomass conversion processes and catalysts for specific chemicals according to the molar ratio of H_2_ to CO. We take the synthesis of ethyl alcohol as an example to introduce the indirect liquefaction process.

### 2.2. Reaction Mechanism

**Figure 3:**
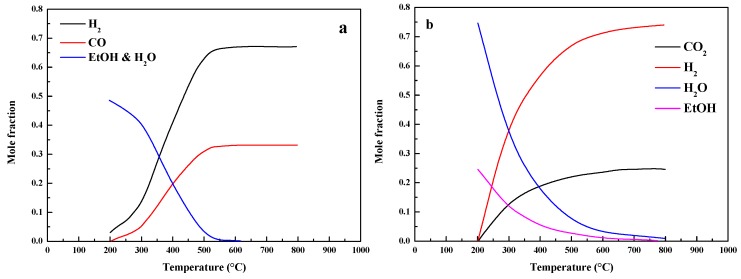
Thermodynamic analysis of the hydrogenation of CO (**a**) and CO_2_ (**b**; H_2_/CO = 2.0, H_2_/CO_2_ = 3.0, 30 bar).

**Figure 4:**
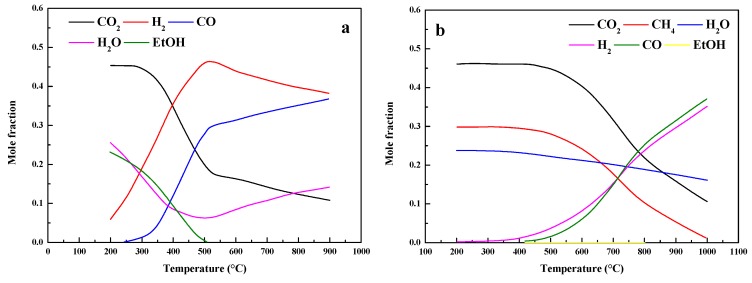
Equilibrium concentrations of mixture syngas: No CH_4_ allowed (**a**) and CH_4_ allowed (**b**; H_2_ = 49%, CO = 26%, CO_2_ = 21%, and H_2_O = 4%).

There are two approaches to produce ethyl alcohol [13]. The first pathway is hydrogenation of CO, and the reaction equation is shown in Equation (1). From the equation, it indicates that hydrogenation of CO is a highly exothermic and favorable reaction. Figure 3a shows the thermodynamic analysis of the hydrogenation of CO, and the reaction conditions (H2/CO = 2.0, 30 bar) are assumed. The results release that the concentrations of ethyl alcohol and water decrease with a temperature increase, while the concentrations of H_2_ and CO increase with a temperature increase. Hence, the suitable reaction temperature should be below 350 °C via the hydrogenation of CO to produce ethyl alcohol. The second pathway is hydrogenation of CO_2_, and the reaction equation is shown in Equation (2). From the equation, it indicates that hydrogenation of CO_2_ is also a highly exothermic and favorable reaction. The thermodynamic analysis of the hydrogenation of CO_2_ is shown in Figure 3b. From the results, the concentrations of ethyl alcohol and water decrease with a temperature increase, while the concentrations of CO_2_ and H_2_ increase with a temperature increase. The reasonable temperature for the synthesis of ethyl alcohol via the hydrogenation of CO_2_ should also be below 300 °C. However, the by-products would be formed during the F–T process. For hydrogenation of CO, the H_2_O product would react with CO quickly, and the by-products of CO_2_ and H_2_ would be formed, which refers to the water gas shift reaction (Equation (3)). On the contrary, the reverse water gas shift reaction may be occurred in the hydrogenation of the CO_2_ process, and the by-product is CO. It indicates that the two pathways proceed through a common intermediate. In addition, the methanation (Equations (4) and (5)) would occur along with the hydrogenation of CO or CO_2_, and CH_4_ is the most significant by-product [14]. Figure 4 shows the equilibrium concentrations of an initial mixture syngas. From the results, it indicates that the suitable temperature should be below 400 °C at 30 bar with no CH_4_ formation. However, if CH4 is allowed as a product under the same conditions, the content of ethyl alcohol is virtually zero. Therefore, CH_4_ must be kinetically limited to improve the yield of ethyl alcohol. Moreover, the equilibrium concentration of ethyl alcohol increases with the reaction pressure increase. As shown in the literature, there are little researches that studied the hydrogenation of CO_2_ or the mixture of CO and CO_2_, and the primary researches studied the hydrogenation of CO. The catalyst is the key factor for this reaction, and it could be divided into four groups, including Rh-based materials, modified CH_3_OH synthesis catalysts, modified F–T catalysts, and modified Mo-based catalysts [15,16].

2CO + 4H_2_ → C_2_H_5_OH, ∆H = −61.2 kcal·mol^−1^, ∆G = −29.3 kcal·mol^−1^, (1)

2CO_2_ + 4H_2_ → C_2_H_5_OH, ∆H = −41.5 kcal·mol^−1^, ∆G = −15.7 kcal·mol^−1^, (2)

CO + H_2_O → CO_2_ + H_2_, (3)

CO_2_ + 4H_2_ → CH_4_ + 2H_2_O, (4)

CO + 4H_2_ → CH_4_ + H_2_O. (5)

## 3. Direct Liquefaction

### 3.1. Hydrolysis-Fermentation Liquefaction

#### 3.1.1. Main Steps

**Figure 5:**
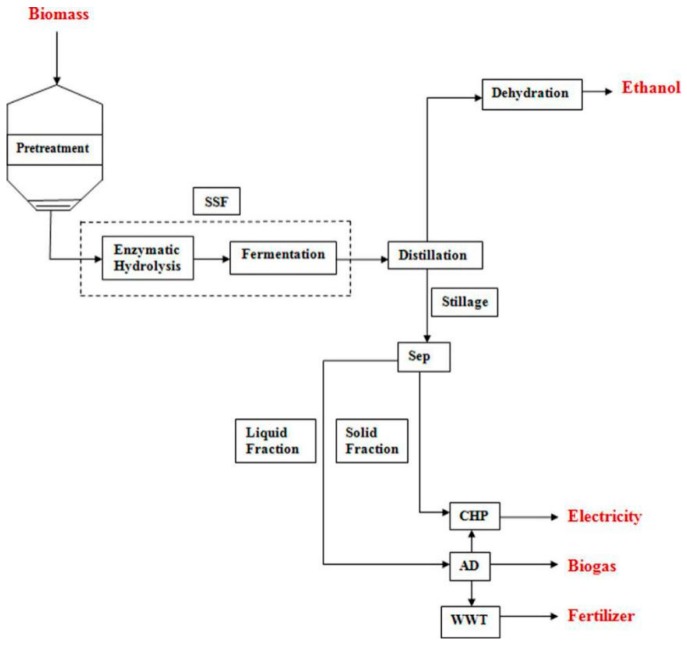
Flow diagram of the enzymatic ethanol production process [21].

In the last few decades, ethyl alcohol has attracted a great deal of attention as a potential alternative to fossil fuels [9]. Currently, fermentation of biomass is the main industrial technology to produce ethyl alcohol, which the primary raw materials are glucose (obtained from corn) and sucrose (obtained from sugar cane and beets) [17]. While, there are the same negative effects on ethyl alcohol production using starch or sugar as the raw material, which would compete with food production directly [18,19]. Up to now, corn straw has been considered as possible raw material for ethyl alcohol production [20]. The flow diagram of enzymatic ethanol production process is shown in Figure 5. Once the biomass is transported to the production plant, it would be stored in the warehouse to prevent from fermentation and bacterial contamination. Then, the raw material would be pre-treated to make it more accessible for extraction. In the fermentation process, hydrolysate, yeasts, nutrients, and other ingredients would be added. The fermentation is usually executed at 25–30 °C and the suitable reaction time would last for 6–72 h. The parameters are primarily dependent on the components of hydrolysate, type, density, or activity of yeasts. The recycle yeasts are employed to improve the activity and productivity of fermentation. The concentration of ethyl alcohol in the broth typically contains 8–14%, while the activity of yeasts would be inhibited above this concentration. After distillation, a mixture may be obtained, which is often termed “hydrous” or “hydrated” ethyl alcohol (95% alcohol, 4% water). The hydrated ethyl alcohol would be then dehydrated to obtain “anhydrous” ethyl alcohol (99.6% alcohol, 0.4% water). The remaining vinasse or stillage from the distillation column could be valorized to produce process steam and electricity, products for feeding animals, fertilizer, and other valuable by-products. During the hydrolysis-fermentation process, pre-treatment has been recognized as a necessary upstream process.

#### 3.1.2. Pre-Treatment

Cellulose in biomass has a straight chain structure composed of dehydrated glucose groups connected β-1,4 glucoside bonds resulting in a high degree of polymerization and stable property [22]. It is difficult to dissolve in water at room temperature, even at high temperature the dissolution is also very slow [23]. The representative pre-treatment includes a physical pre-treatment [24], chemical pre-treatment [25], and biological treatment pre-treatment [26]. The advantages and weaknesses of selected pre-treatment processes are listed in Table 1. Biological pre-treatment is an eco-friendly technology, while the efficiency is very low (residence time: 10–14 d) for industrial purposes [27]. In addition, careful growth conditions, a large amount of space, and a high cost of enzymes make it difficult to attract an entrepreneur’s attention [28]. Physical pre-treatment refers to the reduction of physical size of raw materials to increase enzyme-accessible surface areas [29], and the primary pathways of physical pre-treatments are mechanical comminution [30], pyrolysis [31], and steam explosion [32]. Chemical pre-treatment refers to the process of using chemicals to remove or modify hemicelluloses and lignin. The primary pathways of chemical pre-treatments are acidic pretreatment [25], alkali pre-treatment [33], sulfur dioxide [9], organosolv [34], liquid hot water (LHW) [35], and wet oxidation [36]. Currently, dilute acid pre-treatment, steam explosion, and liquid hot water (LHW) pre-treatment have the best performance.

(1) LHW Pre-Treatment

In the LHW pre-treatment process, the liquid water is used to promote disintegration and separation of lignocellulose, which the water could maintain a liquid phase at high temperature and pressure [9,35]. The typical temperature ranges from 160 °C to 240 °C, and the reaction time ranges from 5 min to 1 h. The parameters are dominated by the types of biomass and sugar formation [37]. During this pre-treatment, no acid and chemical catalysts are required, and there is a high xylose recovery (88–98%) [38]. Although this method is economically interesting and environmentally attractive, it requires higher energy due to high temperature and pressure and a large amount of water [39].

(2) Acidic Pre-Treatment

The most studied method is acidic pre-treatment for biomass, and it could provide the satisfactory cellulose conversion [25]. According to the literature, H_2_SO_4_, HCl, H_3_PO_4_, and HNO_3_ have been studied, and these acids would promote the hydrolysis of hemicelluloses and amorphous cellulose [25,40,41,42]. However, the concentrated acids are not favored due to the corrosion and toxicity in nature. In the industrial scale, dilute acid pre-treatment is more popular. Generally, dilute acid refers to the concentration of 0.5–1.5% [43]. The yield of sugars is high from hemicelluloses. In this pre-treatment process, the lignocellulose could be usually executed at high temperature (about 180 °C) for a shorter period of time (less than 15 min), while the materials could be also performed at low temperature (about 120 °C) for a long reaction time (about 30–90 min) [44]. For instance, the total sugar recovery from poplar wood is about 83% with a loading of 15 FPU/g cellulose [45]. During this process, different simple sugars are released from hydrolysis of hemicellulose, e.g., xylose, arabinose, mannose, and galactose, while it also releases some other compounds, which could inhibit the enzymatic hydrolysis and fermentation. Finally, part of the CH_3_COOH, H_2_SO_4_, and other inhibitors produced during the degradation are removed, and neutralization is formed. However, dilute acid pre-treatment only provides satisfactory enzymatic cellulose conversion for hardwood. Cellulose conversion is only about 40% when using softwood as the raw material [46].

(3) Steam Explosion

Steam explosion is another effective pre-treatment for biomass. During the process, steam would make the biomass reach to the target temperature rapidly without excessive dilution of sugars [32]. Generally, the raw material is treated by hot steam under high pressure, e.g., 180–240 °C, 1–3.5 MPa, and then the lignocellulosic structure would be destroyed [26]. The sudden release of pressure contributes to defibrillation of cellulose bundles, which improves the accessibility of cellulose for enzymatic hydrolysis and fermentation. There are two stages in the steam explosion process: Auto-hydrolysis and de-pressurization [47]. In the auto-hydrolysis process, the hydrolysis of hemicellulose occurs when acetic acid from acetyl groups connected with hemicellulose is formed at high temperature. The acetic acid would further catalyze the hydrolysis of hemicelluloses [48]. Then the particle size of raw materials would be reduced resulting in the high enzymatic accessibility of cellulose in the depressurization process. This method leads to a remarkable breakdown of lignocellulose, hydrolysis of hemicellulose, depolymerization of lignin, accessibility of enzymes [47]. The primary advantages of this method are less hazardous chemicals, high energy efficiency, and low environment pollution. In order to further improve the energy efficiency, some scientists reported that the addition of SO_2_, CO_2_, and NH_3_ would achieve this goal [49,50]. The method also names the acid-catalyzed steam pre-treatment. For instance, the raw materials are impregnated with acid catalyst either in the gas phase with SO_2_ or in the aqueous phase with H_2_SO_4_ before steam pre-treatment. We think that the acid-catalyzed steam pre-treatment is actually another form of the dilute acid pre-treatment. Typical acid or SO_2_ charge on biomass varies from 0% to 5%, and the temperature ranges from 190 to 220 °C. Reaction time varies from 1 to 10 min [49,50]. The addition of NH_3_ (ammonia fiber explosion) would also greatly improve the conversion efficiency [51]. In ammonia fiber explosion, lignocellulose biomass is exposed to liquid ammonia at a moderate temperature (60–100 °C) under a high pressure (1.5–2.0 MPa) for a period of time, and then the pressure is suddenly released [52,53]. However, the important limitation is that hemicellulose would not be removed significantly, leading to a lower enzyme accessibility and yield of sugars.

### 3.2. Thermodynamic Liquefaction

In general, there are two types for thermodynamic liquefaction of biomass depending on the operating conditions: Pyrolysis liquefaction [31] and hydrothermal liquefaction [54]. In pyrolysis liquefaction, it could be divided into slow pyrolysis, fast pyrolysis, and flash pyrolysis [55]. Bio-oil, which is also regarded as pyrolysis oil or pyrolytic oil, could be obtained from both of these two methods. As shown in the literature, bio-oil is the extremely complex substance and composed of hundreds of organic compounds, e.g., alkanes, aromatic hydrocarbons, phenol derivatives, ketones, esters, ethers, sugars, amines, and alcohols [56,57]. In addition, the molar ratio of H to C in bio-oil is higher than 1.5. The pyrolyze bio-oils could be directly burned in boilers, or upgraded to produce valuable fuels and chemicals using the following methods: Extraction [58], emulsification [59], esterification/alcoholysis [60], supercritical fluids [61], hydrotreating [62], catalytic cracking [63], and steam reforming [64].

#### 3.2.1. Fast Pyrolysis

Slow pyrolysis is usually executed at a low reaction temperature, heating rate, and a long residence times, which produces a little of bio-oil. In the flash pyrolysis process, the reaction time is only or less than several seconds with a very high heating rate and small particle size, and the primary product is syngas [65]. Fast pyrolysis also proceeds at a high heating rate (less than in flash pyrolysis) and short residence time of the vapor [66]. The favorable product in the process is bio-oil. This technology has developed considerably in the last years, due to the low investment costs, high energy efficiencies, and environmental acceptability [67,68]. In the fast pyrolysis, the core technology is the reactor design. During the last decades, in order to realize rapid heat-transfer, several different reactor designs have been developed [68,69,70], such as fixed bed, bubbling fluidized bed, circulating fluidized bed, rotating cone reactor, Auger reactor, and vacuum reactor. The choice of the technology and quality of bio-oil are affected by many parameters, including raw material, reaction temperature/time, particle size of raw material, residence time of volatiles, heat transfer rate, and feed rate.

##### Operating Parameters

(1) Raw Material

The main components in biomass are cellulose, lignin, hemicellulose, and inorganic substance, and the percentage of these components is mainly influenced by the biomass species [71]. Even with the same biomass, the percentage of these components is also dependent on soil, age, or planting conditions [72,73]. The percentage of the components affects the yield and distribution of bio-oil. The higher yield of bio-oil would be obtained from the higher contents of cellulose and hemicellulose than that obtained from a higher content of lignin component [74,75]. As the greater structural stability, lignin component is difficult to be decomposed and the primary pyrolysis product is biochar [76]. Quan et al. [77] reported that the yield of bio-oil obtained from cellulose is 18.67%, the yield of bio-oil obtained from hemicellulose is 30.83%, and the yield of bio-oil is only 0.5% using the lignin as the raw material. Stefanidis et al. [78] studied the pyrolysis products using cellulose, hemicellulose, and lignin as the raw material. It indicates that the main liquefaction products of cellulose are sugars, levoglucosan, and low concentrations of phenols, ketones, aldehydes, and alcohols, the main liquefaction products of hemicellulose are ketones, phenols, acids, and aldehydes, and the main liquefaction products of lignin are complex phenols with high molecular weights. Moreover, ash also has a significant effect on the proportion and distribution of fast pyrolysis products [79]. High content of ash component leads to a low yield of bio-oil and high proportions of biochar and gas-phase substance [80]. Especially, Na and K could reduce the yield of liquid-phase products, and S and P could also reduce the yield of bio-oil resulting in the higher biochar formation [80,81]. The content of moisture is another critical factor in the fast pyrolysis process [82]. The production and collection of raw material affects the content of water [83]. The water in bio-oil is the result of the absorbed water in raw materials and dehydration reactions during the pyrolysis process. Generally, the maximum moisture content is 10% in the raw material to minimize the water content in the bio-oil [17].

(2) Reaction Temperature

The reaction temperature is the key factor in the fast pyrolysis process. It could provide the necessary energy for the decomposition of biomass bonds [84]. The decomposition efficiency increases with reaction temperature increase, because the high temperature could provide the high energy to break the biomass bonds [85]. Numerous studies have shown that the high yield of bio-oil generally ranges from 450 °C to 500 °C [86,87,88], while the temperature changes with the raw material or the other variables. Although the high temperature may cause a positive effect on the yield of bio-oil, the extremely high temperature often has the negative impact on the yield, which may occur the secondary cracking of the volatiles [89]. Table 2 lists the suitable temperatures for different biomasses. Besides the yield, the temperature also has a significant effect on the quality of bio-oil. At low temperatures, the main components in bio-oil are alkenes, alkanes, long-chain fatty acids and esters, aliphatic nitriles, and amides, while the components obtained at higher temperature change to short aliphatic carbons and lower-molecular-weight compounds of alcohols, ketones with lower H/C ratio [90,91].

(3) Residence Time

A large amount of vapor would be released during the fast pyrolysis process. After the reaction, the vapor should be removed rapidly from the reactor. If the vapor were not removed in the minimum time, the secondary reactions would occur [74,102], such as thermal cracking, repolymerization, and recondensation of the biochar residue. The secondary reactions would cause a decrease in the yield of bio-oil. In order to purge the pyrolysis vapor, N_2_ is the most popular gas due to being inert, cheap, and readily available. In the process, a higher inert gas flow should be introduced to reduce vapor residence time [103]. Several studies analyzed the effect of inert gas flow on the yield of bio-oil show that the increase in the flow of inert gas causes an improvement in the bio-oil yield (Table 3). However, a very high gas flow would lead to a lower yield of bio-oil, because the uncompleted condensation of vapors and biomass are swept out of the reactor, resulting in a low yield of bio-oil and high yield of the gas-phase products.

(4) Particle Size

As a poor conductor of heat, of biomass, it is difficult to transfer heat into biomass in the pyrolysis process. Hence the yield of bio-oil is dependent on the particle size, and decreasing the particle size is a vital task in minimizing the heat transfer problem [74]. Shen et al. [110] reported that the yield of bio-oil with a 0.3 mm particle size increased by 12–14% compared to that with a 1.5 mm particle size. Garg et al. [86] reported that the yield of bio-oil increases when the particle size of a babool seed decreases in the fixed bed reactor, and the raw material with less than 0.4 mm leads to a maximum yield of bio-oil (32%). Kang et al. [111] observed similar results. However, there are some negative opinions in the effect of particle size. Encinar et al. [94] reported the fast pyrolysis of grape bagasse, *Cynara cardunculus* L. and soybean cake in the fixed bed reactor. The result indicates that it has less effect on the yield of bio-oil when the particle size of the raw material is up to 2 mm. Abnisa et al. [112] believed that the yield of bio-oil is independent of the particle size, and they observed the opposite effect in palm shell pyrolysis. The result indicates that the yield of bio-oil increases to 69.6 wt. % when the particle size increase from 0.5 mm to 2 mm. Generally, it is known that the smaller particle size has the faster and uniform heating resulting in the higher yield. However, the fine raw material would reduce the yield of bio-oil as the uncompleted fine biomass would be removed out of the reactor by inert gas [106].

(5) Other Factors

Reaction time refers to the time sustained in the reactor for the raw material at the specific pyrolysis temperature. In order to obtain the high yield of bio-oil, a sufficient reaction time is required during the pyrolysis process. If the reaction time is too long, the secondary reactions would be occurred for the pyrolysis vapors, e.g., carbonization, gasification, or thermal cracking, resulting in a lower yield of bio-oils [113]. Moreover, this parameter is vital to the reactor design. The reaction time for some biomasses is listed in Table 4. Feed rate is another factor in the fast pyrolysis process that affects the product distribution and quality. The formation of gas and organic vapor occurs under the low feeding rate and fast heat transfer, which contributes to faster liquefaction [7]. However, as the yield of the gas-phase products with low feed rate is lower than that with fast feed rate, the higher residence time is required, which leads to an increase in the yield of biochar due to the long interaction between vapor and biochar. Furthermore, the long residence time could also cause secondary reactions [114].

##### Reactor Types

(1) Bubbling fluidized bed reactor

**Figure 6:**
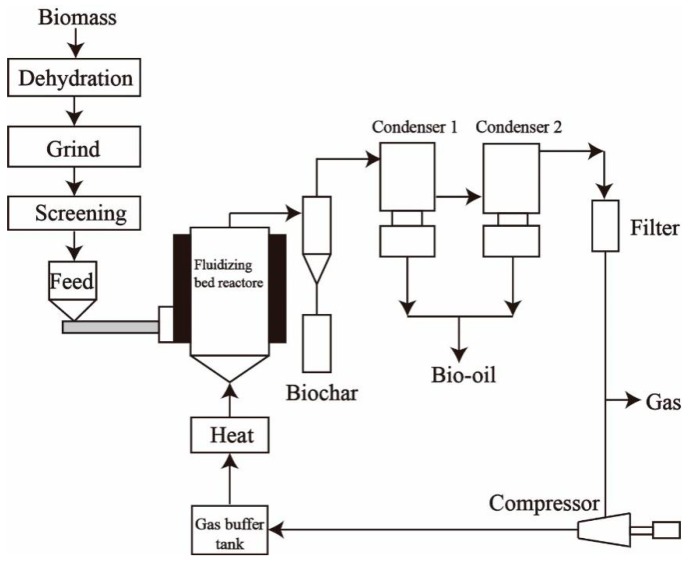
Schematic for the bubbling fluidized bed.

As is well known, the bubbling fluidized bed reactor has become popular in the petrochemical industry for decades [119]. The reactor design could provide a high heat transfer rate and uniform bed temperature in the fast pyrolysis process of biomass. Generally, the residence time in the freeboard section above the bed ranges from 0.5 to 2.0 s dependent on the size of the bed fluidizing media [120]. The reaction temperature is usually set to 500–550 °C resulting in the highest yield of bio-oil, while the temperature and residence time could be operated at a low level in the larger systems. The parameters are also influenced by the type of raw material. During the reaction, the yield of bio-oil would be oxidized using the flue gases as the direct heating media. The small particle size (2–3 mm) is required to reach the good heat transfer. Self-cleaning is an advantage of this reactor with a relatively narrow particle size distribution, as the biochar (by-product) would be carried out with the gas and vapor. If the biomass particle size is too large, the biochar could not be entrained out of the reactor and then the lower density of biochar would stick on the top of the bed [121]. Figure 6 shows the schematic of a typical bubbling fluidized bed [122,123]. The biochar on the top of the bed would act as the role of the catalyst to converse the vapors when they pass through the biochar, resulting in the lower yield of bio-oil. If the size of the raw material is too fine, it must be introduced lower in the bed to prevent the fine feed from entraining out of the bed before complete pyrolysis. Hence, a mean should be designed for skimming and discharging biochar from the top of the bed. If it is not to be designed, the raw material should carefully grind to obtain a narrow particle size distribution leading to a high cost. Otherwise, heat applied to the reactor in different ways should be also designed to improve the flexibility of the reactor.

(2) Circulating fluidized bed

**Figure 7:**
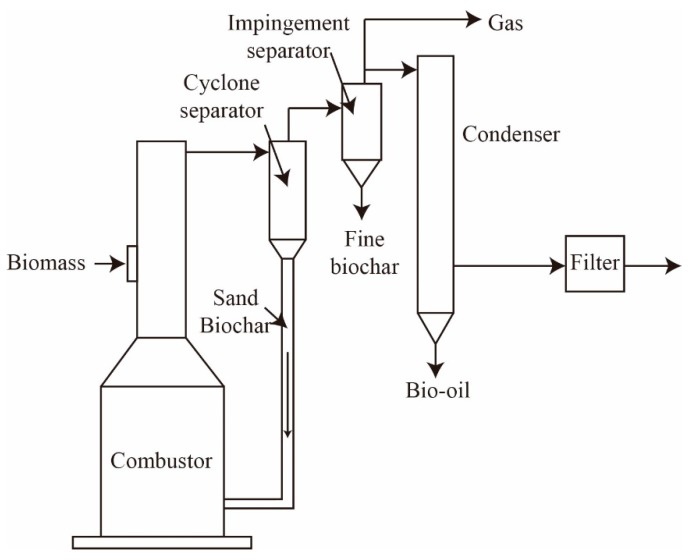
Schematic for the circulating fluidized bed.

Circulating fluidized bed technology has been extensively applied to biomass pyrolysis. The advantage of the reactor is high heat transfer rates and short vapor residence times [124]. Figure 7 shows the schematic of this circulating fluidized bed system [125,126]. In the design, large quantities of sand should be moved into the reactor resulting in the high challenge of the operability. The hot sand could be circulated between combustor and pyrolyzer units. The most important difference is the supplying heat method for various developed system designs. The first generation contains the simple units, e.g., single indirectly heated reactor, cyclone, and standpipe configuration. In this system, the particle size of the raw material should be smaller than that in bubbling beds, and the residence time would only have 0.5–1.0 s (s) in the high heat transfer pyrolysis zone [127]. For the large particle size of the raw material, there is not enough time to transfer the heat. Especially, biochar is formed on the surface of biomass, which plays a vital role to prevent further penetration of heat. The incomplete pyrolyzed larger particles would be burned in the char combustor with a low yield of bio-oil. Hence, the suitable particle size is 1–2 mm [128].

(3) Rotating cone reactor

**Figure 8:**
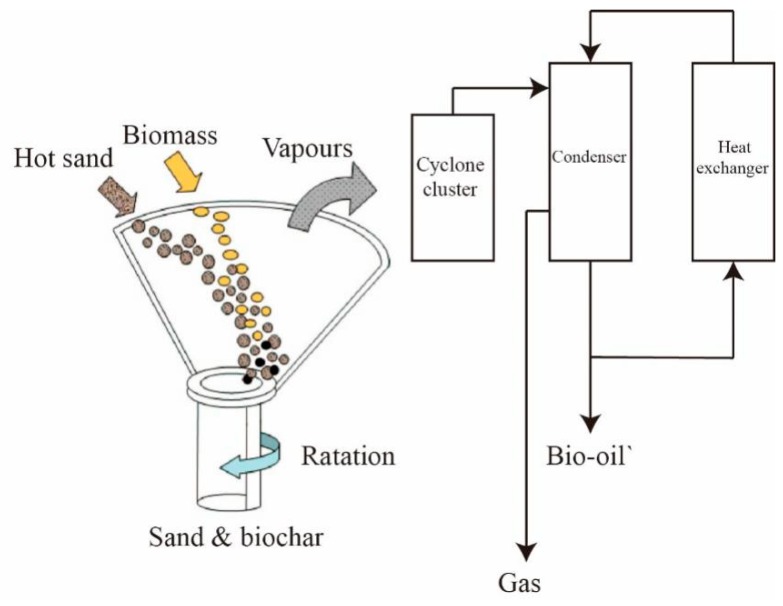
Schematic for the rotating cone reactor.

The rotating cone reactor has been in development since the early 1990s, and the processing capacity has been improved to 200 kg/h. The schematic for the rotating cone reactor [129] is shown in Figure 8. Similar to the circulated fluidized bed, the pyrolysis reaction executes with the mixture of hot sand and biomass. While, the primary distinction between them is that the carrier gas is replaced by the rotary cone to form a centrifugal force. Firstly, the raw material and sand are introduced to the base of the rotating cone, and then the mixture is moved to the lip of the cone under a centrifugal force. Finally, the mixture spills over the lip of the cone, and the pyrolysis vapor would be introduced to the condenser. The sand would be re-heated after introducing biochar and sand into the combustor at the base of the cone. The literature demonstrates that the yield of bio-oil would achieve to a 70% yield and there are two advantages in this design. The first is that the bio-oil would recover easier without the carrier gas, and the second is that the wear problems are reduced because the transport dynamics of sand and biomass are not as aggressive as in the circulating fluid bed. However, the disadvantages are a complex integrated process and scale up issue [130].

(4) Auger reactor

**Figure 9:**
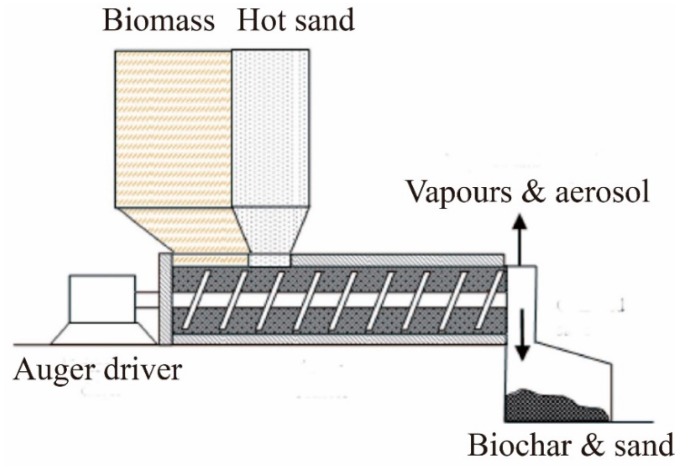
Schematic for the Auger reactor.

The important advantage of an Auger reactor has been identified for its potential to reduce operating costs [131]. However, this reactor is not suitable for large-scale pyrolysis [132]. Figure 9 shows a schematic for the Auger reactor. In the reactor, the Auger system plays the role of the commingler for the hot sand and raw material. Generally, the design contains hot, heating, and circulation systems. Once the raw material is introduced into the reactor, it would direct contact with a bulk solid heat transfer medium. The materials of the heat transfer medium are usually sand or a steel shot, which is heated independently before sending into the reactor. According to the gravimetric basis, it is suggested that the feed rate of the heat transfer medium is 20 times to that of the raw material. It is effective when the biomass and heat transfer medium are combined with two intermeshing, co-rotating augers quickly in a shallow bed during the pyrolysis reactions. This is an unintelligible mechanical mixing process. Finally, the vapors and aerosols are introduced out of the different ports respectively, and biochar would be transported through a long section and stored in a canister with the heat transfer medium [133,134].

(5) Vacuum pyrolysis

**Figure 10:**
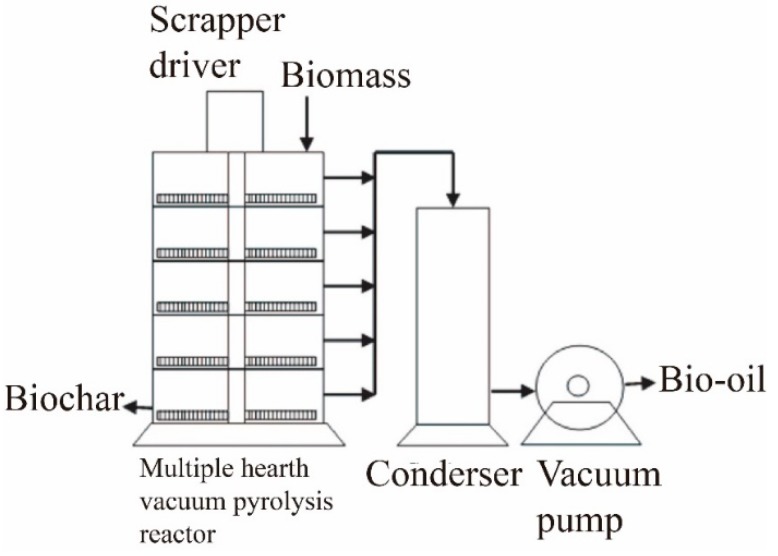
Schematic for the vacuum pyrolysis reactor.

The vacuum pyrolysis process has been developed by Canada. In fact, vacuum pyrolysis belongs to the slow pyrolysis process. The low heat transfer rate and short vapor residence time would produce the bio-oil product [135]. The yield of bio-oil is only half of that obtained by the fluid bed technologies [136]. Figure 10 shows the schematic design of vacuum pyrolysis. In this design, the raw material is moved by gravity and rotating scrappers through multiple hearth pyrolyzers, and the reaction temperature changes from 200 °C to 400 °C. Furthermore, a larger particle size and a little carrier gas are required. The raw material would be carried into the high temperature vacuum chamber using a moving metal belt. The biomass on the belt is stirred by the mechanical agitator periodically. All of this mechanical transport is being done at 500 °C. The special devices of feeding and discharging are required to achieve a good seal at all times. However, the high investment and maintenance cost limit its development [137,138].

#### 3.2.2. Hydrothermal Liquefaction

##### Hydrothermal Processing

According to the literature, the content of water in the biomass raw material is usually more than 40% for pyrolysis liquefaction [139]. Hence, it is usually required to pre-treat the biomass to make it suitable for pyrolysis application. The reported pre-treatments are atmospheric drying, solar drying, and evaporation [140,141]. Few studies suggest that the water could be mechanically dehydrated via atmospheric drying. Although the cost of the solar drying method is low, it requires a long dehydrated time to lower the water content [142,143]. The presence of water in the raw material could consume more energy to evaporate it, which limits the operability and economy of this technology [140,144]. Compared to the pyrolysis liquefaction, hydrothermal liquefaction of biomass is one of the effective methods to treat the biomass with high water content [54,144]. This liquefaction of biomass is not affected by the level of water content and the types of biomass with high conversion and relatively pure products [145]. The suitable properties for liquefaction of biomass are demonstrated [146], including a high density, good heat, mass transfer capability, fast decomposition, and extraction under hydrothermal conditions. This is an environment friendly technology, and the heteroatom in biomass could be converted into undesired by-products. Biomass and O element would be rapidly oxidized to form CO_2_ or H_2_O [142]. N element in biomass is mainly converted to N_2_ or N_2_O. S, Cl, and P elements are mainly oxidized to their inorganic acids, which would destroy the vessel lining [147]. Hence, extreme operating conditions, corrosion, and scaling are major limitations of hydrothermal liquefaction.

##### Operating Parameters

(1) Temperature

It is known that the yield of bio-oil and the biomass fragmentations would be increased with the temperature increase [145,148]. The depolymerization of biomass would occur extensively when the reaction temperature is higher than the activation energies for the bond cessation. However, the high temperature increases not only the concentration of free radicals, but also the probability of repolymerization of fragmented species [140,149]. The suitable reaction temperature for bio-oil production is the competitive result of hydrolysis, fragmentation, and repolymerization. At the initial stage, depolymerization of biomass is the dominant reaction, and the biochar would be formed via repolymerization at the later stage [150]. Hence, the higher yield of bio-oil would be obtained at the intermediate temperature. As the properties of water would change rapidly under a super-critical condition, it is a huge challenge to select the optimum temperature [151]. The yield of bio-oil is influenced by the reaction temperature sequentially. The reaction temperatures for bio-oil production via hydrothermal liquefaction of some biomass are listed in Table 5. Generally, when the temperature is lower than 374 °C, the yield of bio-oil increases with the reaction temperature. While, the gas-phase products would increase when the temperature is higher than 374 °C. Furthermore, it is not suitable to produce bio-oil at a high temperature considering the operational cost and bio-oil yield. A large amount of gas-phase products would be formed via secondary decompositions and Bourdard gas reactions, and formation of biochar would be produced due to the recombination of a high concentration of free radical [152,153]. In addition, the yield of bio-oil would be suppressed by the incomplete decomposition of components when the temperature is lower than 280 °C. Hence, the effective temperature range would be located from 300 °C to 350 °C for hydrothermal liquefaction of biomass.

(2) Pressure

In general, pressure is another key factor for the hydrothermal liquefaction of biomass. The single-phase solvent would be maintained under the high pressure, and the large heat is required to maintain the energy of the reaction system for the two-phase system [158]. When the pressure of the system is higher than the critical pressure of the solvent, the favorable reaction pathway would be enhanced for the production of liquid fuels. On the other hand, the solvent density would be improved under the high pressure, and the molecules of biomass components could be penetrated by the high density solvent, which enhances the pyrolysis and extraction. While, there is little influence on the yield of bio-oil when the reaction pressure overcomes the supercritical conditions for liquefaction [153,154].

(3) Residence time

The residence time is also the key factor for the hydrothermal liquefaction of biomass, and the products components and conversion of biomass would be defined by the residence time [157]. In the supercritical conditions, the reaction rate of hydrolysis and decomposition is rapid, so the short reaction time is contribution to the decomposition of the raw material [159]. However, the reaction time is required to optimize the destruction of the organic compounds in different biomasses [160]. Different researches have studied the function of reaction time on the yield of bio-oil. Boocock et al. [161] reported that the yield of bio-oil decreases for a long residence time except for a very high mass ratio of biomass to water. Generally, the breakage of C–C bands leads to the depolymerization that biomass depends upon. However, the cage effect for C–C bands would inhibit C–C bonds breakage under the supercritical pressures, resulting in a low fragmentation.

(4) Other parameters

Firstly, the higher heating rate would promote the fragmentation of biomass and inhibit the formation of biochar, while there is a low effect on the product distributions [162]. The reason for the phenomenon is that the fragmented species would be dissolved and stabilized in the supercritical conditions [156,162]. Secondly, the particle size of raw material also affects the yield of bio-oil. In order to achieve the high-efficiency hydrothermal liquefaction, reducing the particle size of raw material would increase its accessibility [156]. While, the treatment would improve the consumption of energy resulting in the increase of cost. Hence, the hydrothermal liquefaction should be executed with an optimum particle size at a low grinding cost. In fact, it has a low effect in hydrothermal liquefaction for the particle size of biomass [163]. The sub/supercritical water acts as not only a heat transfer medium but also an extractant, and it could overcome the heat transfer limitations. Therefore, the particle size of the biomass is less important, it is not needed for excessive size reduction of the raw material. Thirdly, there is the potential effect of the solvent type and density on the yield of bio-oil in hydrothermal liquefaction. The common solvents in the reported researches are water, methyl alcohol, ethyl alcohol, and other organic solvents [148]. For solvent density, it is a key factor for the mass ratio of solvent to biomass. The high mass ratio of solvent to biomass contributes to the high yield of bio-oil and gas products, which is because the products would be exacted by the solvent medium [162]. Wang et al. [164] reported that the high mass ratio of solvent to biomass could increase the amount of bio-oil, meanwhile there is a decrease in the yields of residues and gas-phase products. It indicates that the solvent could enhance the stability and solubility of fragmented components. When the mass ratio of biomass to solvent is high, the relative interactions between biomass components and the solvent become weak, leading to a low dissolution capacity of biomass components.

#### 3.2.3. Upgrading of Bio-Oil

##### Bio-Oil Characteristics

Bio-oil is the dark brown and viscous liquid with a distinctive odor [66,92]. Bio-oil is a complex mixture of several hundreds of chemical composition, and affected by the type of raw material, thermochemical process, and operating parameters [91,141]. The main components in bio-oil are acids, alcohols, aldehydes, esters, ketones, phenols, and guaiacol [55,165]. The undesirable properties of bio-oil are caused by these compounds. The physical characteristics of liquefaction-derived bio-oil [59,166,167,168] are listed in Table 6. As shown in the table, the concentrations of water and the O element in bio-oil are higher than that in heavy petroleum fuel oil, resulting in a lower heating value of bio-oil (about 35 MJ/kg). In addition, the concentration of the N element in bio-oil is also higher than that in heavy petroleum fuel oil. The pH value of bio-oil ranges from 3.5–4.2 leading to a high corrosivity. For instance, the acidity and corrosiveness is the result of a considerable amount of fatty acids. Moreover, the side reactions would proceed during the storage period, e.g., polymerization and evaporation would cause a slow increase in viscosity [90,169]. Besides, aldehydes are the most unstable component in bio-oil. The above undesired characteristics of bio-oil have limited its application as a transportation fuel. Hence, it is necessary to upgrade the quality of bio-oil.

##### Bio-Oil Upgrading

Reported by the literature, there are physical and chemical methods to upgrade bio-oil, including extraction, solvent addition, emulsification, esterification/alcoholysis, supercritical fluids (SCFs), hydrotreating, catalytic cracking, and steam reforming. Table 7 lists the process conditions, advantages, and disadvantages for each method [58,59,60,61,62,63].

(1) Extraction

As listed above, there are a large number of chemicals in bio-oil, and some of the chemicals have industrial applications, e.g., phenol for resin industry, organic acids, and alkanes [170]. Therefore, the chemicals extracted from bio-oil are effective in increasing the value of bio-oil. Similar to petrochemical plants, extraction from bio-oil is the separation technology [171]. The technology includes absorption, distillation, and fractionation. The solvent for adsorption is acetone, and the fractionation is divided into phase separation and aqueous extraction [172]. In Eboibi et al.’s work [173], they reported that there is a decrease in the concentration of the O element, and an increase in HHV. In order for commercialization, the low-cost separation and refining techniques must be developed [174].

(2) Solvent addition

Using polar solvents to reduce viscosity of bio-oils has been popularized for several decades, and the common polar solvents include ethyl acetate, acetone, methanol, and ethanol [175,176]. After polar solvents addition, the heating value increases as the solvents has the higher heating value than bio-oil [177]. On the other hand, the viscosity of bio-oil would be reduced because there is a physical dilution and chemical reaction between solvent and bio-oil components.

(3) Emulsification

Emulsification with other chemicals is another method for bio-oil upgrading. While, it is difficult to mix bio-oil derived from biomass and fuels derived from petroleum, so the surfactants are commonly used [178]. The ignition property of bio-oil emulsions is excellent, while higher corrosion is observed in engine applications. Emulsification is a short-term pathway to upgrade bio-oil without a chemical reaction [59]. Although, this pathway could improve the ignition characteristics, there is no significant improvement in heating value, corrosiveness, and cetane number [179]. Emulsification is a relatively simple technique to upgrade bio-oil with diesel or bio-diesel, however the addition of surfactants and the high energy consumption result in a high cost [180].

(4) Esterification/alcoholysis

In order to obtain bio-diesel, esterification (also named alcoholysis) of free fatty acids to alkyl ester is popular [181]. Acid catalyst plays an important role in the process for the reaction between fatty acids and alcohol to produce alkyl ester or bio-diesel under atmospheric pressure [182]. Due to the low cost, the most common alcohol is methanol for esterification. The reaction is usually carried out at a lower temperature (<60 °C), which is lower than the boiling point of methanol. The esterification reaction could also be performed under super-critical conditions. The super-critical upgrading of bio-oil is more effective than sub-critical upgrading. However, there is plenty of water formation in the reaction as the by-product. Esterification is a reversible reaction, and water could be separated and removed to increase the yield after reactive distillation. Although the catalysts for esterification could be divided into homogeneous and heterogeneous, the easier separation from the products have been preferred to apply in bio-oil upgrading, and the popular catalysts are HZSM-5 and aluminum silicate. Besides fatty acids, there is also the esterification reaction for aldehydes to produce acetal. Some characteristics of bio-oil have been improved after esterification and acetalization, such as viscosity, density, aging rate, acidity, oxygen content, and water content, leading to a high heating value. However, there is no literature that reported significant nitrogen removing. In brief, the advantages of esterification are simplicity, low temperature, pressure, and cost of alcohols, which seems to be one of the promising techniques for upgrading bio-oil [183].

(5) Supercritical fluids (SCFs)

A supercritical fluid is a new method for upgrading bio-oil and has attracted a lot of attention. Beyond the critical point of the solvent, the solvent would maintain a fluid at a high temperature and pressure [184], which refers to the super-critical fluid, and it is difficult to distinguish the liquid phase and gas phase [185]. The material could be dissolved in the super-critical fluid, while it could also diffuse through solids like a gas [186]. It is a promising alternative to organic solvents. In the supercritical fluid, the ability of undissolved materials could be improved [187]. The unique properties of the technology are liquid-like density, gas-like diffusivity and viscosity, and faster rates of mass and heat transfer. According to the literature [188,189,190,191], some solvents have been studied to upgrade bio-oil, including ethanol, methanol, and water. It is similar to esterification under super-critical conditions using alcohol as a solvent and acid catalyst as a catalyst [192]. Xu et al. reported that the properties of bio-oil would be improved using supercritical 1-butanol on Ru/C catalyst [193]. Duan et al. [194] reported the upgrading of bio-oil at high H_2_ pressure under a supercritical condition with a Pt/C catalyst. After treatment, the higher heating value of bio-oil increases, and acid number, O and N contents, and viscosity decrease.

(6) Hydrotreating

It is generally recognized that the higher content of hydrogen in the fuel product leads to a better quality [184]. Hydrotreating of bio-oil is an established process to reduce the concentrations of O, S, and N, and this technology would be executed with a catalytic reaction under high pressure hydrogen (10–20 MPa) at moderate temperatures (250–450 °C) [195]. O element is removed in the form of water, N element and S element are also removed in the form of NH_3_ and H_2_S respectively [196]. The primary methods for hydrotreating technology are hydrogenation and hydrodeoxygenation [65].

For hydrogenation, it aims to improve the quality and stability of bio-oil via reducing the contents of reactive compounds, e.g., organic acids and aldehydes [197,198]. The traditional technology for hydrogenation of bio-oil is single, which is carried out at specific conditions, e.g., high hydrogen pressure, high temperature, and suitable catalysts [14,199]. The catalysts used in the hydrogenation of bio-oil are usually Al_2_O_3_-based catalysts and the Ru/SBA-15 catalyst. After upgrading, all of the pH value, water content, and H element concentration increase, while the viscosity of bio-oil decreases to some extent [194,200]. Currently, the new hydrogenation method names one-step hydrogenation-esterification (OHE). In OHE, acids and aldehydes could be converted to stable and combustible substances, and the common catalysts are bifunctional, e.g., Pd-Al-SBA-15 catalyst [201,202]. The bifunctional catalysts could offer the abilities to upgrade bio-oil via hydrogenation and esterification. Yu et al. [203] reported that 5 wt. % Pd@Al_2_(SiO_3_)_3_ exhibits a catalytic performance, and it could convert the unstable components in bio-oil to esters or alcohols via the effective OHE reaction. Tang et al. [204] also reported similar results. Besides, the catalytic performance for bifunctional catalyst has been improved via some treatments, and the OHE method is much better than the traditional method [205].

For hydrodeoxygenation (HDO), it is a variant of hydrogenation upgrading, and the O element in many kinds of oxygenated chemical groups could be removed under high hydrogen pressure, e.g., acids, aldehydes, esters, ketones, and phenols [169,206]. The common catalysts in previous literature for HDO focused on Ni-Mo or Co-Mo sulfide/supported hydrotreating catalysts (Table S1). Wang et al. [207] reported that the mesoporous Pt/ZSM-5 exhibits a more excellent performance than Pt/ZSM-5 and Pt/Al_2_O_3_. However, there are typical disadvantages for the HDO, including by-product formation, catalyst deactivation, and a high cost for noble metal. Hence, the novel and economical catalysts should be developed that contains a high oxygen content. In China, the largest scale reactor is a 500 mL autoclave reactor with a 10 mm diameter and 420 mm length. Recently, the amorphous catalysts have attracted the attentions as the excellent hydrodeoxygenation activity and selectivity. Amorphous Co-W-B catalyst shows an excellent catalytic hydrodeoxygenation ability [208,209]. Simple preparation, high stability and activity, and a low cost make the amorphous catalyst as the potential candidate for HDO.

(7) Catalytic cracking

Catalytic cracking could be divided into two pathways to improve the quality of bio-oil: One is traditional catalytic cracking and another one is the combination of catalytic pyrolysis and catalytic cracking [63]. The traditional catalytic cracking means that bio-oil suffers a thermal conversion treatment at a higher temperature with high pressure hydrogen flow using the proper catalysts in a tubular fixed bed reactor. There are solid (coke), liquid, and combustible gases products after the catalytic cracking process [210]. The liquid products could be divided into the organic phase and aqueous phase. The common catalyst in this reaction is HZSM-5 [63], while the bottleneck for sustainable application of catalysts is the coke deposition of this catalyst [211]. The combination of catalytic pyrolysis and catalytic cracking is the integration of catalytic pyrolysis and catalytic cracking. The reactor design is divided into a traditional pyrolysis reactor and a decomposition of gaseous intermediate apparatus. According to the literature, the length is 1.0 m with an inner diameter of 20 mm for the biggest reactor design using a 316 stainless tube [72]. The combined technology has the superiority of improving the yield of bio-oil and the quality of fuel. Complementally, hydro-cracking is the less popular catalytic cracking process with an external hydrogen source. The reaction temperature is usually greater than 350 °C, and the reaction pressure exceeds 2000 psi. During the reaction, the complex organic substances are cracked into simpler molecules. Currently, the C–C bands are broken by hydrogen [212]. Moreover, it is popular for the combination of hydrotreating and hydro-cracking for upgrading bio-oil. In the combination, the hydrotreating of bio-oil is carried out firstly and then heavy components in the bio-oil are broken into light components after the hydro-cracking process [213,214]. Although hydro-cracking is an effective method to break heavy components in bio-oil, the high reaction temperature and hydrogen pressure are required resulting in a high cost [215].

(8) Steam reforming

Steam reforming refers to a technology to produce synthesis gas from fossil fuels, and the target products are hydrogen and carbon monoxide [216]. During the reaction, the liquid fuel is usually reacted with a high temperature steam. The steam reforming of bio-oil is the extension of the steam reforming of fossil fuels, and the target products are also syngases [217]. The technology is investigated in a fixed bed reactor and luidized bed reactor [218]. The reaction temperature, steam-to-carbon ratio, and catalyst-to-feed ratio play important roles in steam reforming of bio-oil, and the most common catalysts in steam reforming of bio-oil are Ni-based catalysts [219,220]. Similar to hydrotreating, the bottleneck for the sustainable application of catalysts is also the coke deposition of these catalysts [221].

## 4. Conclusion and Recommendations for Future Work

### 4.1. Conclusion

Fossil fuels may run out in several decades, and the renewable fuels (nuclear energy, solar energy, wind energy, and biomass energy) should be further developed. Biomass has the potential to be used on a large scale. The liquefaction of biomass is an important technology to converse the biomass into valuable biofuel. Direct liquefaction of biomass is promising technology to converse the biomass into biofuels, and the main methods are hydrolysis fermentation and thermodynamic liquefaction. Bio-oil could be upgraded by physicochemical methods.

### 4.2. Recommendations for Future Work

(1) As we know that the components of biomass are very complex, and the types of biomass are also various. These would be the key factors to affect the liquefaction process. Thus the liquefaction method should be selected based on the type of biomass.

(2) Considering the indirect liquefaction, conversion CO_2_/CO and H_2_ to ethyl alcohol is a potential technology for biomass utilization. In this process, the catalysts should be developed to achieve efficient conversion.

(3) For the hydrolysis-fermentation liquefaction, this is a traditional technique. While the primary issue is the long reaction period. The more efficient engineering bacteria should be explored based on genetic technology.

(4) The liquid fuel (bio-oil) obtained from fast pyrolysis and hydrothermal liquefaction is the potential mixture to replace fossil fuels. However, the high concentrations of water and the O element in bio-oil limited its application. Further work should focus on the hydrotreating upgrading.

## Figures and Tables

**Table 1:** Advantages and weaknesses of selected pretreatment processes.

Pretreatment		Yield of FS *	Chemical Recycling	Wastes	Investment
Physical	Mechanical	-	++	++	+
Physico-chemical	Steam explosion	+	++	+	-
	Ammonia fiber explosion	+/-	--	+	-
	Carbonic Acid	++	++	++	+
Chemical	Dilute acid	++	--	-	+/-
	Concentrated acid	++	--	-	-
	Alkaline extraction	++/+	--	-	++
	Wet oxidation	+/-	++	+	+
	Organosolv	++	--	+	--

* FS: Fermentable sugars; ** ++: very good; +: good; -: bad; --: very bad.

**Table 2:** Suitable temperatures for different biomasses.

Raw Material	Temperature	Yield	Ref.
Rice husk	450	70 wt. %	[91]
Plam	500	72.4	[92]
Neem deoiled cake	400	40.2	[93]
*Cynara cardunculus* L.	400	46.23	[94]
Olive bagasse	600	46.3	[95]
Sugarcane bagasse	475	56	[96]
Cassava rhizome	472	63.23	[97]
Cassava stalk	472	61.39	[97]
Jatropha seed shell cake	470	48	[98]
Poplar	455	69	[99]
Pistachio shell	550	20.5	[100]
Bamboo sawdust	510	61	[101]

**Table 3:** Suitable residence time for different biomasses.

Raw Material	Gas Flow Rate/Time	Yield of Bio-Oil	Ref
Safflower seed	100 cm, 3/min	67 wt. %	[104]
Palm kernel shell	1 L/min	50 wt. %	[7]
	1.5 L/min	53 wt. %	
	2 L/min	57 wt. %	
Sewage sludge	300 mL/min	45.3 wt. %	[6]
Rice husk	3 L/min	45 wt. %	[102]
	4 L/min	47.5 wt. %	
	5 L/min	49 wt. %	
Cassava stalk	0.1 L/min	48 wt. %	[105]
	0.5 L/min	51 wt. %	
	1.5 L/min	53 wt. %	
	3 L/min	52 wt. %	
Sugarcane bagasse	5 s (8 L/min)	56+1.3 wt. %	[106]
	10 s (4 L/min)	52+2.5 wt. %	
	20 s (2 L/min)	47.5+28 wt. %	
Jatropha cake	1.25 m, 3/h	37.78 wt. %	[107]
	1.75 m, 3/h	64.25 wt. %	
	2.4 m, 3/h	30.5 wt. %	
Babool seeds	100 cm, 3/min	44 wt. %	[86]
	400 cm, 3/min	30 wt. %	
Rice husk	0.255 m/s	19.5 wt. %	[108]
	0.340 m/s	20.5 wt. %	
	0.425 m/s	17.9 wt. %	
Euphorbia rigida	400 cm, 3/min	31.5 wt. %	[109]
Sunflower pressed bagasse	200 cm, 3/min	45.7 wt. %	
Hazelnut shells	100 cm, 3/min	23.1 wt. %	

**Table 4:** Suitable reaction time for different biomasses.

Raw Material	Reaction Time/min	Yield of Bio-Oil	Ref
Rice husk	1	36 wt. %	[5]
	2	41 wt. %	
	4	40 wt. %	
	8	39 wt. %	
Rice straw	1	9 wt. %	[115]
	2	10 wt. %	
	4	9.5 wt. %	
	8	8 wt. %	
Bagasse	1	7 wt. %	
	2	16 wt. %	
	4	11 wt. %	
	8	10 wt. %	
Coconut shell	1	5 wt. %	
	2	13 wt. %	
	4	7.5 wt. %	
	8	11 wt. %	
Pistachio shell	10	52.96 wt. %	[116]
	20	53.08 wt. %	
	50	50.13 wt. %	
Physic nut	15	27 wt. %	[117]
	240	22.5 wt. %	
	30	28 wt. %	[118]
	60	46 wt. %	
	90	45.5 wt. %	
	120	45 wt. %	
	150	45.8 wt. %	
Cassava stalk	60	52 wt. %	[4]
	180	39.5 wt. %	
Cassava rhizome	60	50 wt. %	
	180	42 wt. %	

**Table 5:** Reaction temperatures via hydrothermal liquefaction for some biomasses.

Raw Material	Temperature	Yield	Ref.
*Enteromorpha prolifa*	300	21	[154]
Cattle manure	315	38	[155]
Grassland perennials	300	77	[156]
Eucalyptus	305	36	[151]
*Cunninghamia lanceolata*	305	76	[157]
*Dunaliella tertiolecta*	360	22	[152]

**Table 6:** Physical characteristics of liquefaction-derived bio-oil and heavy petroleum fuel oil.

Properties	Bio-Oil	Heavy Petroleum Fuel Oil
pH	3.8–4.0	-
Acid value (mgKOH/g)	1.8	-
Density (g/cm3)	1150–1200 at 40 °C	940
Viscosity (cP)	650 at 40 °C	180 at 40 °C
HHV (MJ/kg)	28.42	40
C (wt. %)	66	85
H (wt. %)	11	11
O (wt. %)	12	1.0
N (wt. %)	9	0.3
S (wt. %)	1	-
Water content (wt. %)	13–12	0.1
Ash content (wt. %)	0.4–0.7	0.1

**Table 7:** Brief description of bio-oil upgrading techniques.

Upgrading Techniques	Process Conditions	Pros.	Cons.
Extraction	Mild conditions, solvents	Extracts valuable chemicals from bio-oil	Low cost separation and refining techniques are still needed
Solvent addition	Mild conditions, polar solvents	Simple	No chemical reaction to convert or remove undesired compound within bio-oil
Emulsification	Mild conditions, surfactant	Simple	High energy consumption, no chemical reaction to convert or remove undesired
Esterification/alcoholysis	Mild conditions, alcohol	Relatively simple, mild conditions, low cost of alcohol if methanol is used	Not effective to remove nitrogen-containing compounds
Supercritical fluids (SCFs)	Relatively high pressure and temperature, organic solvents	Effective to increase HHV and reduce viscosity	Needs high pressure equipment, some solvents are expensive
Hydrotreating	Relatively high pressure and temperature, catalysts	Removes N, O, and S as NH_3_, H_2_O, and H_2_S, and increase HHV, commercialized already	Needs high pressure equipment, high cocking and catalyst deactivation
Catalytic cracking	Relatively high temperature, atmospheric pressure, catalysts,	Produces large amounts of light products	Needs high pressure equipment, catalyst deactivation
Steam reforming	High temperature, catalyst	Produces H_2_ as a clean energy resource	Needs high temperature equipment

## Supplementary Materials

The following are available online, Table S1: Catalysts for hydrodeoxygenation.
