# Supplementary material for: Liquefaction of Biomass and Upgrading of Bio-Oil: A Review
Source: Molecules. 2019 Jun 17;24(12):2250. doi: 10.3390/molecules24122250 (PMC6630481; doi:10.3390/molecules24122250)
Supplement: Supplementary file 1 [file molecules-24-02250-s001.pdf]

Table S1 Catalysts for hydrodeoxygenation

Active site	Support	Temperature	Component	Products	Conversion	Ref.
MoO_3	-	400	Acetone	Propylene	96.8	65[1]
			Cyclohexanone	Pentenes	100	
MoC_2	-	360	m-cresol	Benzene	90	66[2]
NiMo	Al_2O_3	450	Acetic acid	Ethanol	65	67[3]
Pt	γ-Al_2O_3	300	Guaiacol	phenol	-	72[4]
Pt	HBETA SiO_2	400	Anisole	benzene	90	73[5]
Pt	Al_2O_3	300	Guaiacol	Anisole	70	74[6]
Pt	γ-Al_2O_3	300	m-cresol	Toluene	74	78[7]
Fe	SiO_2	400	Guaiacol	Benzene	77	79[8]
Fe	SiO_2, AB	400	Guaiacol	Benzene	100	80[9]
Ga	HBETA SiO_2	400	m-cresol	toluene	80	82[10]
Ru	TiO_2	400	Pyrolysis vapors			83[11]
W	Carbon	380	propanol/propanal	propene	-	84[12]
Ni	Al_2O_3	260	1-octanol	Octenes	87.4	85[13]
Zn	Al_2O_3	550	Bio-oil		Benzene	86[14]
Cu	Carbon	250-450	Guaiacol	Toluene	65	87[15]
Ni_2P	SiO_2	300	Guaiacol	Phenol	100	93[16]

References:

[1]. T. Prasomsri, T. Nimmanwudipong, Y. Roman-Leshkov, Effective hydrodeoxygenation of biomass-derived oxygenates into unsaturated hydrocarbons by MoO_3 using low H_2 pressures, Energy Environ. Sci. 6 (6) (2013) 1732–1738.

[2]. Chen, C., W. Lee and A. Bhan, Mo2C catalyzed vapor phase hydrodeoxygenation of lignin-derived phenolic compound mixtures to aromatics under ambient pressure. Applied Catalysis A: General, 2016. 510: p. 42-48.

[3]. N. Joshi, A. Lawal, Hydrodeoxygenation of acetic acid in a microreactor, Chem. Eng. Sci. 84 (2012) 761–771

[4].Nimmanwudipong, T., et al., Catalytic Conversion of Guaiacol Catalyzed by Platinum Supported on Alumina: Reaction Network Including Hydrodeoxygenation Reactions. Energy & Fuels, 2011. 25(8): p. 3417-3427.

[5]. Zhu, X., et al., Bifunctional transalkylation and hydrodeoxygenation of anisole over a Pt/HBeta catalyst. Journal of Catalysis, 2011. 281(1): p. 21-29.

[6]. Nimmanwudipong, T., et al., Catalytic Reactions of Guaiacol: Reaction Network and Evidence of Oxygen Removal in Reactions with Hydrogen. Catalysis Letters, 2011. 141(6): p. 779-783.

[7]. Zanuttini, M.S., et al., Deoxygenation of m-cresol on Pt/ γ -Al2O3 catalysts. Catalysis Today, 2013. 213: p. 9-17.

[8]. Olcese, R.N., et al., Hydrodeoxygenation of Guaiacol, A Surrogate of Lignin Pyrolysis Vapors, Over Iron Based Catalysts: Kinetics and Modeling of the Lignin to Aromatics Integrated Process. Energy & Fuels, 2013. 27(2): p. 975-984.

[9]. Olcese, R., et al., Gas-phase hydrodeoxygenation of guaiacol over iron-based catalysts. Effect of gases composition, iron load and supports (silica and activated carbon). Applied Catalysis B: Environmental, 2013. 129: p. 528-538.

[10]. Ausavasukhi, A., et al., Hydrodeoxygenation of m-cresol over gallium-modified beta zeolite catalysts. Journal of Catalysis, 2012. 290: p. 90-100.

[11]. Wan, S., et al., Direct catalytic upgrading of biomass pyrolysis vapors by a dual function Ru/TiO2 catalyst. AIChE Journal, 2013. 59(7): p. 2275-2285.

[12]. Ren, H., et al., Tungsten carbides as selective deoxygenation catalysts: experimental and computational studies of converting C3 oxygenates to propene. Green Chem, 2014. 16(2): p. 761-769.

[13]. Chandra Sekhar Palla, V., D. Shee and S.K. Maity, Kinetics of hydrodeoxygenation of octanol over supported nickel catalysts: a mechanistic study. RSC Adv., 2014. 4(78): p. 41612-41621.

[14] S. Karnjanakom, A. Bayu, X. Hao, S. Kongparakul, C. Samart, A. Abudula, G. Guan,Selectively catalytic upgrading of bio-oil to aromatic hydrocarbons over Zn, Ce or Ni-doped mesoporous rod-like alumina catalysts, J. Mol. Catal. A Chem. 421(2016) 235–244,

[15] J. Sun, A.M. Karim, H. Zhang, L. Kovarik, X.S. Li, A.J. Hensley, J.S. McEwen,Y. Wang, Carbon-supported bimetallic Pd-Fe catalysts for vapor-phase hydrodeoxygenation

of guaiacol, J. Catal. 306 (2013) 47–57
[16] P. Bui, J.A. Cecilia, S.T. Oyama, A. Takagaki, A. Infantes-Molina, H. Zhao, D. Li, E. Rodríguez-Castellón, A. Jiménez López, Studies of the synthesis of transition metal phosphides and their activity in the hydrodeoxygenation of a biofuel model compound, J. Catal. 294 (2012) 184–198
